# Obstetric Neuropathy in Diabetic Patients: The “Double Hit Hypothesis”

**DOI:** 10.3390/ijms24076812

**Published:** 2023-04-06

**Authors:** Dieu Thao Nguyen, Mohammad Hooshmand Zaferanieh, Asa C. Black, Kamron Reza Hamedi, Richard L. Goodwin, Thomas I. Nathaniel

**Affiliations:** Greenville School of Medicine, University of South Carolina, 607 Grove Road, Greenville, SC 29605, USA

**Keywords:** diabetic neuropathy, neurological damage during delivery

## Abstract

The two-hit model has been proposed to explain the effects of diabetes on mothers who are already in a putative subclinical damaged state and then undergo neuronal damage during the delivery process. However, the anatomical and pathophysiological mechanisms are not well understood. Our overarching hypothesis in this review paper is that pregnant women who are diabetic have a damaged peripheral nervous system, constituting the “first hit” hypothesis. The delivery process itself—the “second hit”—can produce neurological damage to the mother. Women with diabetes mellitus (DM) are at risk for neurological damage during both hits, but the cumulative effects of both “hits” pose a greater risk of neurological damage and pathophysiological changes during delivery. In our analysis, we introduce the different steps of our concept paper. Subsequently, we describe each of the topics. First, we outline the mechanisms by which diabetes acts as a detrimental variable in neuropathy by focusing on the most common form of diabetic neuropathy, diabetic distal symmetrical polyneuropathy, also known as distal sensorimotor neuropathy. The possible role of macrosomia in causing diabetic neuropathy and obstetric neurological injury is discussed. Second, we describe how vaginal delivery can cause various obstetrical neurological syndromes and pathophysiological changes. Third, we highlight the risk of obstetric neuropathy and discuss anatomical sites at which lesions may occur, including lesions during delivery. Fourth, we characterize the pathophysiological pathways involved in the causation of diabetic neuropathy. Finally, we highlight diabetic damage to sensory vs. motor nerves, including how hyperglycemia causes different types of damage depending on the location of nerve cell bodies.

## 1. Introduction

Diabetes mellitus (DM) describes a state of chronic hyperglycemia that affects more than 460 million individuals worldwide [1]. The number of patients with DM has been projected to rise to 700 million worldwide by 2045 [2]. Two types of diabetes mellitus are currently recognized based on their etiologies: Type 1 and Type 2. Type 1 DM describes an autoimmune disease process in which pancreatic beta cells have been targeted for destruction; therefore, there is insufficient insulin production, leading to hyperglycemia. Type 2 DM has been characterized as hyperglycemia due to insulin resistance. Patients with Type 2 DM produce insulin; however, the body does not respond appropriately [1].

Diabetic distal symmetrical polyneuropathy, or distal symmetrical sensorimotor neuropathy (DSSPN), is the most common form of diabetic neuropathy [3,4]. It is responsible for foot lesions, including ulcers and amputations [5]. The lesions manifest in a “stocking and glove” pattern. This form of diabetic neuropathy begins by damaging the sensory axons of the feet first and then the hands. Thus, the symptoms are correlated with the length of the axon. This form disturbs sensory function first and then damages the autonomic nervous system and somatic motor function. Pre-diabetic conditions exhibit a similar pattern [6]. Chronic hyperglycemia damages Schwann cells, which are responsible for the myelination of axons in the peripheral nervous system. It has been noted that there are features of demyelination in patients with severe cases of hyperglycemia. The current literature has only determined that Wallerian degeneration is involved in signaling pathways that induce axonal degeneration [4].

## 2. The Mechanisms of Diabetic Neuropathies Are Complex

**Figure 1:**
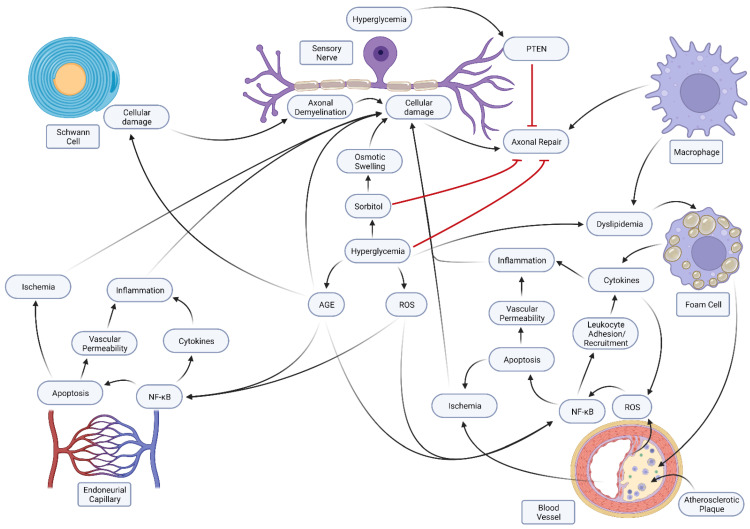
Pathways Involved in diabetic neuropathy: hyperglycemia causes diabetic neuropathy through several pathways. Elevated blood glucose leads to an increased conversion of glucose to sorbitol. Sorbitol can cause osmotic swelling, causing compression neuropathy, and can inhibit axonal repair mechanisms. Hyperglycemia can also cause direct damage to nerve cells through the stimulation of the PTEN (phosphatase and tensin homolog) pathway. Hyperglycemia causes Schwann cell toxicity, thereby producing advanced glycation end products (AGEs) and leading to axonal demyelination. Macrophages are important for nerve repair and regeneration, but can be inhibited by hyperglycemia. Hyperglycemia also leads to dyslipidemia and the formation of foam cells from macrophages. These foam cells contribute to the formation of atherosclerotic plaques, leading to vascular and endoneurial microvascular damage to produce reactive oxygen species (ROS). ROS use the NF-κB pathways to cause leukocyte recruitment and apoptosis, leading to inflammation, ischemia, and cellular nerve damage [16].

The pathogenesis of diabetic neuropathy is complex [7]. Diabetic distal symmetrical polyneuropathy (DSP) is a length-dependent polyneuropathy. It is generally considered to be multi-factorial. DSP is considered to involve interactions between glycemic control, the duration of diabetes, and neuronal attrition related to age, hypertension, and body weight. Alterations seen in DSP include segmental demyelination, microangiopathy, and Wallerian degeneration. Apoptosis of dorsal root ganglia is an important mechanistic consideration that causes the loss of unmyelinated and myelinated fibers. Entrapment neuropathies are also common in the diabetic population, affecting one in every three patients [8,9]. Dellon [10] suggested three mechanisms that cause diabetic patients to be more susceptible to peripheral neuropathy. The osmotic hypothesis [11] postulates that there is an increase in the conversion of glucose to sorbitol in the diabetic population. Sorbitol has hydrophilic characteristics, which increase water concentration in the nerves, causing osmotic swelling. It has been shown that the median and tibial nerves in patients with diabetes have significantly larger cross-sectional areas than in a healthy, non-diabetic control group [12]. The swelling in a defined area, such as the carpal or tarsal tunnel, can lead to compressive neuropathy (Figure 1). Second, an abnormal metabolic state and high sorbitol concentration in diabetic patients can impair the nerve regeneration pathway [13] (Figure 1). Lastly, hyperglycemia can produce high amounts of advanced glycation end products (AGE) that can bind to collagen in the peripheral nerve and nearby ligaments, altering the biomechanical AGE properties of both the nerve and ligament. The hyperglycemic state is an additional mechanism that can cause compressive neuropathy when swelling occurs in a restricted space [9,13]. A different concept involves the role of inflammation and vascular complications, especially concerning pain [14]. Vascular injury in diabetes is often mediated by the development of atherosclerosis (Figure 1). Hyperglycemia leads to dyslipidemias, encouraging the formation of foam cells. These cells contribute to the buildup of plaques in the blood vessels. These foam cells also release cytokines that stimulate endothelial cells to produce reactive oxygen species (ROS). ROS use the NF-κB pathways to cause leukocyte recruitment and apoptosis, leading to inflammation, ischemia, and cellular nerve damage [15].

Diabetes mellitus targets the axons of sensory neurons, autonomic nervous system neurons, and motor neurons [4]. Schwann cells are damaged in hyperglycemia, and in more severe cases, demyelination of the axons occurs, which is reflected in the stocking-glove pattern of damage (Figure 1). The axons of the neurons supplying the feet are longer than those supplying the hand, so the most significant damage appears first in the feet (length-dependent demyelination) [4]. Considering the role of Schwann cells in the maintenance and repair of axons, damage to Schwann cells may play a key role in the failure of axons to be maintained or repaired.

**Figure 2:**
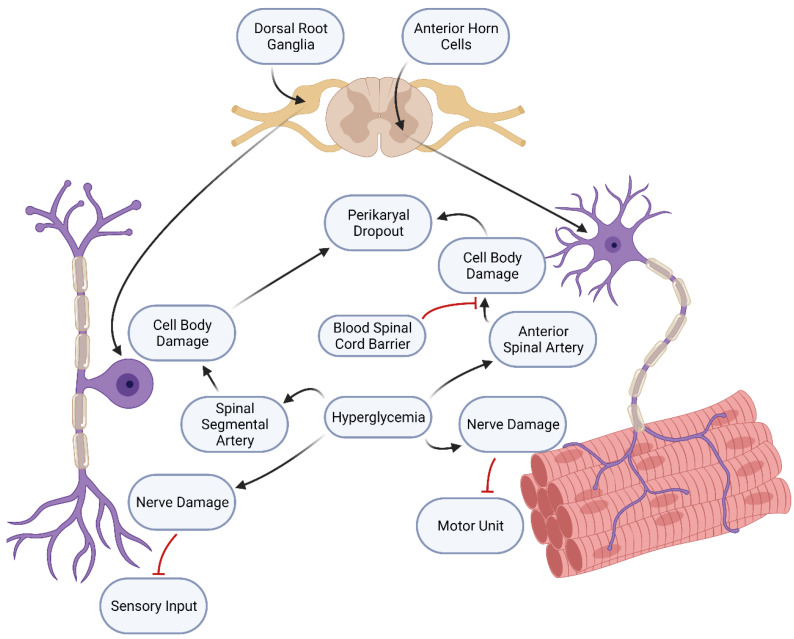
Diabetic damage to sensory vs. motor nerves: hyperglycemia causes different types of damage, depending on the location of nerve cell bodies. Sensory nerve cell bodies are located in the dorsal root ganglia, which are supplied by spinal segmental arteries. Hyperglycemia in this blood supply causes axonal damage and cell body damage, leading to a loss of sensory input and perikaryal dropout. Motor nerve cell bodies are located in the anterior horns of the spinal cord. The blood–spinal cord barrier, similar to the blood–brain barrier, protects these cell bodies from the damaging effects of hyperglycemia. However, the axons of motor nerves and their motor units can still be damaged, leading to the loss of motor function.

Diabetic neurons have an elevated expression of PTEN [17]. PTEN expression inhibits the transduction pathways that are activated by growth factors and required for neuronal regeneration [17,18]. PTEN gene expression persists after injury, thereby interfering with neuronal regeneration. Inhibiting PTEN removes the roadblock to regeneration [18]. The effects on sensory and motor neurons differ somewhat, modeling how general diabetic neuropathy usually affects the functions of sensory neurons. Sensory neuron losses occur first in diabetic mice [19], whose dorsal root ganglia exhibit an increased expression of PTEN compared to littermate controls. This causes neuronal cell death in sensory neurons by disrupting their repair processes. Motor neurons exhibit a distal loss of axon terminals but no perikaryal dropouts (Figure 2). In diabetic mice, the result is the survival of motor neurons but the loss of motor units and neuromuscular junctions, producing a loss of motor function. It has been postulated that the differences between the sensory and motor neurons in terms of the blood–brain barrier can explain the relative vulnerability of sensory neurons compared to motor neurons (Figure 2). The cell bodies of sensory neurons are located in the dorsal root ganglia. In contrast, the cell bodies of motor neurons are located in the anterior horn of the spinal cord. The blood–neuron interface is less well-protected in the dorsal root ganglion than in the spinal cord [20,21,22]. It is noteworthy to point out that surgical decompression can be an effective treatment for diabetic neuropathy [23].

Macrophages may play a role in the axonal damage associated with diabetes mellitus. Associated delays in macrophage invasion and the removal of macrophages have been noted in diabetic mice (Figure 1). This raises the possibility that abnormalities in macrophage participation in axonal regeneration following injury may be deficient or defective since macrophage invasion and removal are different in diabetic mice compared to littermate controls [24]. Alterations in energy metabolism caused by diabetes mellitus may play a role in diabetic neuropathy. The substrate overload in diabetes mellitus leads to the formation of acylcarnitines, which are toxic to Schwann cells and dorsal root ganglion cells [5]. Microcirculatory changes may also damage neurons since the dysfunction of the microcirculatory system is strongly associated with diabetic neuropathy [25]. In this concept, damage to the microcirculation leads to neuronal damage. The pathways that damage this microcirculation are similar to those in other diabetes-associated vasculitides [26]. Endoneurial capillary density is increased in diabetic patients, and findings in rats support this concept [4]. Central nervous system (CNS) changes may also occur, increasing sensitivity to pain [27].

### 2.1. Macrosomic Babies Play a Role in Causing Diabetic Neuropathy and Obstetric Neurological Injury

Gestational diabetes mellitus (GDM) occurs when a previously non-diabetic woman becomes pregnant and develops diabetes mellitus during her pregnancy. Pregnancy places metabolic stress on the mother and can contribute to a state of insulin resistance. As a result, the pancreas will have to produce more insulin to compensate for the resistance of cells to bring in glucose. When the pancreas cannot produce enough insulin, the mother will be hyperglycemic, resembling Type 2 DM [1]. It is noted that approximately 4–14% of pregnancies have complications related to GDM [27].

**Figure 3:**
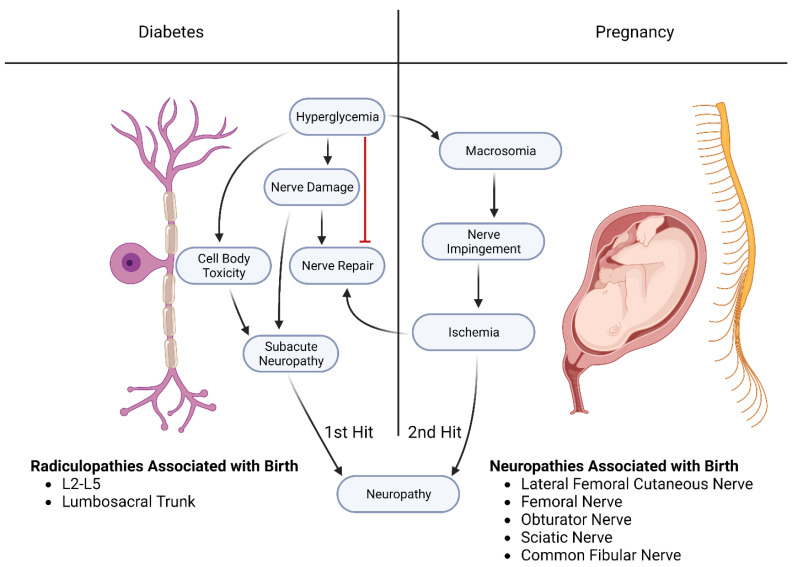
Two-hit hypothesis of diabetic neuropathy in pregnancy: The first hit is believed to be caused by the pathways previously discussed, leading to cell body toxicity and nerve damage. This damage may lead to subacute neuropathy with few symptoms. Diabetes is a common cause of macrosomia, which increases the risk of compression neuropathies during vaginal birth. This compression damage, combined with impaired nerve repair pathways, leads to the second hit, causing the more frequent or severe radiculopathies and neuropathies seen in vaginal births with diabetic mothers.

Diabetes mellitus, coupled with the delivery of a macrosomic baby (>4000 gm) in a short (five feet or less in height) mother, increases the risk of postpartum femoral neuropathy [28]. Diabetes during the gestational period is a known risk factor for causing the development of macrosomic babies, which are risk factors for obstetric radiculopathy, plexopathy, or neuropathy due to cephalopelvic disproportion [29] (Figure 3). The mechanisms that precede fetal macrosomia have been shown to originate from maternal glycemic impairment. High blood glucose levels can travel from the mother’s placenta into fetal circulation. Thus, by the second trimester, the fetal pancreas can respond by releasing insulin to combat the hyperglycemic state, leading to hyperinsulinemia in the fetus. The coupling of the effects of hyperinsulinemia and hyperglycemia can lead to increased fat and protein storage in the fetus and ultimately produce a macrosomic fetus [30].

### 2.2. Mechanism of Injury for Obstetric Lower Extremity Neuropathies

#### 2.2.1. Vaginal Delivery can Cause Various obstetrical Neurological Syndromes

Several clinical neurological conditions are observed during the delivery process and puerperium. These conditions are sometimes referred to as postpartum or intrapartum obstetric neuropathies (sometimes plexopathies) and involve the pelvis and lower limb of the patient. It is estimated that the incidence of such conditions is around 1% of all births [31,32,33]. Specific types include femoral neuropathy, lumbosacral plexopathy, lateral femoral cutaneous neuropathy, common fibular neuropathy, deep fibular neuropathy, and radiculopathy. In addition, pregnancy can exacerbate the development of neuropathies in women with insulin-dependent diabetes mellitus [33].

#### 2.2.2. Obstetric Lesions of Direct Branches of the Lumbosacral Plexus

A lesion of the lateral femoral cutaneous nerve beneath the inguinal ligament is integral to obstetric neuropathy at parturition [34]. Furthermore, this is one of the most common lesions in the delivery process (38% of obstetric lesions [32,33]).

The femoral nerve is a major motor and sensory nerve that also passes beneath the inguinal ligament. When the patient is in the dorsal lithotomy position for hours, excessively abducting and flexing the hips, the inguinal ligament compresses the nerve as it does the lateral femoral cutaneous nerve [35]. An obstetric femoral neuropathy causes significant difficulty in trying to walk. Obstetric femoral neuropathy accounts for 35% of obstetric lesions [33,35].

Two other neuropathies may arise, including (1) obturator nerve neuropathy and (2) sciatic nerve neuropathy. A lesion of the obturator nerve in the delivery process accounts for 5% of cases [32,33]. The sciatic nerve is a major posterior nerve of the lumbosacral plexus. It passes out of the pelvis and divides into the common fibular and tibial divisions. These then pass into the popliteal fossa to form the common fibular and tibial nerves. The mechanism of injury of the sciatic nerve in delivery is uncertain. A lesion of the sciatic nerve properly accounts for 3% of obstetric lesions [32,33]. One proposed mechanism leading to the irritation of this nerve may be the prolonged spasming of adjacent musculatures, such as the piriformis muscle [36,37]. Sometimes the common fibular division of the sciatic nerve passes through the piriformis muscle. When the muscle becomes spastic, pain may be felt along the distribution of the common fibular nerve [38].

#### 2.2.3. Obstetric Lesions of Nerves That Supply the Leg and Foot

The sciatic nerve forms the common fibular nerve and the tibial nerve. The common fibular nerve passes around the neck of the fibula, where it is subject to injury compression. The common fibular nerve is at risk in obstetrical deliveries. When a woman lies in the dorsal lithotomy position, she has the thigh bent (flexed) at the hip and the legs bent (flexed) at the knee. This position puts pressure on the common fibular nerve. Sometimes the patient or an attendant tries to hold the lower limbs in this position for hours during delivery. These actions can cause compression of the common fibular nerve and the obstruction of its blood supply [39,40]. Damage to the nerve can cause pain and numbness due to hyperactivity of the small-diameter pain fibers and hypoactivity of the sensory fibers that supply fine touch to the distribution area. The destruction of the large-diameter motor fibers causes a loss of blood supply and death to the axon at the point of compression. The most significant result is the presence of a foot drop. Common fibular nerve lesions account for 5% of obstetric lesions [32,33].

## 3. Obstetrical Neuropathies

### 3.1. Compressive Neuropathies

In compression neuropathy, a structure such as the patient’s hand or the baby’s head compresses the nerve, obstructing its blood supply. The resulting anoxia damages the axons of the nerve. The larger fibers have a higher oxygen demand and die first. These fibers mediate delicate touch. The smaller fibers mediate pain and temperature sensation. They take longer to die. The pain fibers may become hyperactive and cause pain [41]. The axons die at the point of injury, continuing down the segments distal to the lesion. This is termed Wallerian degeneration [42]. These changes have been studied using magnetic resonance neurography [43]. Because the cell body in the dorsal root ganglion is still viable, the axons will regenerate at a rate of 1–2 mm/day. The peripheral nervous system is noted for its ability to undergo repair. The critical role of Schwann cells in repairing injured axons makes them a prerequisite for nerve repair [19,44,45]. Injury causes Schwann cells to de-differentiate, allowing them to initiate the activity of genes that underlie the repair process. Unfortunately, aging and prolonged injury can limit the ability of Schwann cells to initiate repair [46].

### 3.2. Diabetes-Potentiated Compression Injuries

The specific mechanism involved in the role of diabetes in postpartum neuropathy is not well understood. Compared to matched controls (4.8%), pregnancies among patients with Type 1 diabetes exhibit a 10-fold increase in the prevalence of postpartum neuropathy [31]. These diabetic individuals may have pre-existing (subacute) nerve damage. The subacute injury has yet to manifest clinically; however, further insult can lead to the overt manifestation of symptoms. This could represent anatomical sites already at high risk of either nerve compression or nerve entrapment [32]. Additionally, diabetes-potentiated compression injuries have previously been documented to occur with an increased incidence involving the lateral femoral cutaneous, peroneal (fibular) nerves, ulnar nerve, and median nerve [10]. It has also been suggested that diabetes may increase the risk of femoral nerve compression [33]. Given this predisposition, the pre-existing damage may become more severe when obstetric nerve damage occurs following the delivery process. Such occurrences highlight the importance of further inquiry into the involvement of diabetes in postpartum neuropathy.

## 4. How Diabetes Mellitus Produces Nerve Damage That can Be Potentiated by Any Injury Occurring during Delivery

### 4.1. Hypothesis: Diabetes Mellitus Produces Nerve Damage That can Be Potentiated by Any Injury Occurring during Delivery

One possibility is that parturition can potentiate nerve damage acquired from the diabetic disease process through a double-hit model. The double-hit syndrome can be observed with the first insult coming from the effects of diabetes [10], the mechanisms of which have been outlined above. Subsequently, a second compressive insult may take place as a result of a variety of factors. This could range from the prolonged placement of the patient’s legs in the lithotomy position to the positioning of the fetal head and cephalopelvic disproportion [30], as stated above. The additive consequences of the delivery process can further potentiate damage to a nerve that is already impaired by diabetes. This theory can be limiting as it implies compression is the only cause of nerve neuropathy. However, aside from compression alone, different, multifaceted disease processes contribute to a nerve’s susceptibility to damage.

#### 4.1.1. Diabetes Mellitus Produces a More Severe Neuropathy through a Two-Fold Pathological Process

First, the neurons of diabetics are injured directly by the disease process. The second hit occurs during the attempt to repair the injured neurons, when the regeneration process is subject to molecular blockade [17]. This process involves the PTEN (phosphatase and tensin homolog) gene. PTEN regulates axonal growth by inhibiting the transduction pathway through the P13K pathway, preventing neuronal regeneration. Following an axonal injury, as in diabetes, PTEN expression continues, causing an ongoing regenerative block (second hit) [17]. The regeneration of peripheral nerves is impaired in diabetic mice in which diabetes was induced by treatment with streptozotocin [47]. Considerable delays of up to 8–10 weeks were observed in the regrowth of myelinated axons in diabetic mice [46,47].

#### 4.1.2. Support for the Two-Hit Hypothesis

The concept that neurological injury can be divided into a two-step (“hit”) process has been advanced in different ways. The first example of a two-hit process involves the concept that damage to a neuron by diabetes (“first hit”) can then interfere with the repair process in the damaged neuron (“second hit”). The “first hit” in this concept involves the effects of diabetes mellitus in altering the neurons themselves. In this model, the first hit is the diabetic damage to the neuron that causes it to express elevated levels of PTEN (see above). The “second hit” involves the expression of PTEN, which damages the regenerative capacity of sensory neurons first and motor neurons afterward [14]. Mature neurons, such as those exposed to diabetes mellitus, have a diminished capacity for regeneration [17], which may involve the downregulation of receptors for growth factors.

#### 4.1.3. A Second Example of a Two-Hit Process Involves the Neuronal Damage Caused by Diabetes

In this concept, the first hit involves the effects of diabetes on the axons of the median nerve in the carpal tunnel. The carpal tunnel is a “choke point” in which stressors such as diabetes, pregnancy, and overuse syndromes can damage the axons of the median nerve, causing pain in the distal branches of the median nerve. If the effects are severe enough, motor disturbances can cause loss of function and even atrophy of the thenar muscles of the thumb. Carpal tunnel syndrome is a compressive injury that affects the diabetic population three times more often than the normal population. Although up to one-third of the diabetic population may have carpal tunnel syndrome confirmed through electrophysiological studies, only about 5.8% of these patients will present with clinical symptoms [10]. Therefore, it is likely that diabetic patients may experience different neuropathies, much like that of the axons of the median nerve, without observable symptoms. However, these preceding subclinical neurological conditions can exacerbate the injury during delivery. The two-hit model we have proposed can explain the effects of diabetes on mothers who are already in a putative subclinical damaged state [10] and then undergo neuronal damage during the delivery process.

## 5. Summary and Conclusions

In this review paper, we discussed how the two-hit model can explain the effects of diabetes on mothers who are already in a putative subclinical damaged state and then undergo neuronal damage during the delivery process. In our review, we discussed how pregnant women who are diabetic have a damaged nervous system, although the condition may be subclinical. This constitutes the “first hit”. The delivery process may cause damage to the nervous system of the mother during delivery. This constitutes the “second hit”. Our hypothesis is that pregnant women who have diabetes mellitus are at risk for neurological damage during both hits, but the cumulative effects of both “hits” poses a greater risk of neurological damage during delivery. To support our hypothesis, we discussed how diabetes mellitus produces a more severe neuropathy through different pathological processes. We conclude that diabetic pregnant patients who may have clinical or subclinical neuropathies can acquire a second insult during the delivery process that can exacerbate the presentation of a previously damaged nerve, resulting in symptoms that could interfere with their ability to carry out daily activities and maintain their quality of life.

## Data Availability

Not applicable.
